# Analysis of Plasma Proteins Involved in Inflammation, Immune Response/Complement System, and Blood Coagulation upon Admission of COVID-19 Patients to Hospital May Help to Predict the Prognosis of the Disease

**DOI:** 10.3390/cells12121601

**Published:** 2023-06-10

**Authors:** Daniele Castro di Flora, Aline Dionizio, Heloisa Aparecida Barbosa Silva Pereira, Thais Francini Garbieri, Larissa Tercilia Grizzo, Thiago José Dionisio, Aline de Lima Leite, Licia C. Silva-Costa, Nathalia Rabelo Buzalaf, Fernanda Navas Reis, Virginia Bodelão Richini Pereira, Deborah Maciel Cavalcanti Rosa, Carlos Ferreira dos Santos, Marília Afonso Rabelo Buzalaf

**Affiliations:** 1 Department of Biological Sciences, Bauru School of Dentistry, University of São Paulo, Bauru 17012-901, Brazil; dani_diflora@usp.br (D.C.d.F.); alinesdionizio@usp.br (A.D.); bioheloap@gmail.com (H.A.B.S.P.); tfgarbieri@alumni.usp.br (T.F.G.); larissagrizzo@fob.usp.br (L.T.G.); thiagoj@usp.br (T.J.D.); nathaliabuzalaf@hotmail.com (N.R.B.); fernandanreis@usp.br (F.N.R.); cfsantos@fob.usp.br (C.F.d.S.); 2 Therapy and Diagnosis Unit, Bauru State Hospital, Bauru 17033-360, Brazil; assessoriahospitalar@famesp.org.br; 3 Nebraska Center for Integrated Biomolecular Communication, University of Nebraska-Lincoln, Lincoln, NE 68503, USA; lima.gbm@gmail.com; 4 Laboratory of Neuroproteomics, Institute of Biology, Department of Biochemistry and Tissue Biology, University of Campinas, Campinas 13083-862, Brazil; licinhacosta@gmail.com; 5 Center of Regional Laboratories II, Adolfo Lutz Institute, Bauru 17015-110, Brazil; virichini@yahoo.com.br

**Keywords:** COVID-19, proteomics, biomarker, plasma, prognosis

## Abstract

The development of new approaches allowing for the early assessment of COVID-19 cases that are likely to become critical and the discovery of new therapeutic targets are urgently required. In this prospective cohort study, we performed proteomic and laboratory profiling of plasma from 163 COVID-19 patients admitted to Bauru State Hospital (Brazil) between 4 May 2020 and 4 July 2020. Plasma samples were collected upon admission for routine laboratory analyses and shotgun quantitative label-free proteomics. Based on the course of the disease, the patients were divided into three groups: (a) mild (*n* = 76) and (b) severe (*n* = 56) symptoms, whose patients were discharged without or with admission to an intensive care unit (ICU), respectively, and (c) critical (*n* = 31), a group consisting of patients who died after admission to an ICU. Based on our data, potential therapies for COVID-19 should target proteins involved in inflammation, the immune response and complement system, and blood coagulation. Other proteins that could potentially be employed in therapies against COVID-19 but that so far have not been associated with the disease are CD5L, VDBP, A1BG, C4BPA, PGLYRP2, SERPINC1, and APOH. Targeting these proteins’ pathways might constitute potential new therapies or biomarkers of prognosis of the disease.

## 1. Introduction

In December 2019, in Wuhan, China, several patients were diagnosed with pneumonia caused by a new beta-coronavirus, which was initially called 2019-nCoV and later given the official name severe acute respiratory syndrome coronavirus 2 (SARS-CoV-2) (Coronaviridae Study Group of the International Committee on Taxonomy of 2020). The virus rapidly spread across the globe, initiating an unprecedented pandemic. Within nearly 3 years, about 632 million individuals were infected, leading to nearly 6.5 million deaths globally [1].

Coronaviruses are composed of structural proteins, a core capsid (N), a membrane (M), an envelope (E), and spike (S) protein. SARS-CoV-2 enters human cells using the ACE2 (angiotensin-converting enzyme 2) protein as a receptor. This protein is expressed in the cardiac, respiratory, and gastrointestinal tracts, and it plays a role in regulating the renin–angiotensin system (RAS). The release of a soluble form of ACE2 from the cell surface is regulated by membrane-bound enzymes, such as TMPRSS2 and ADAM17. The enzymatic cleavage of the ACE2 extracellular domain by TMPRSS2 after the binding of the SARS-CoV-2 S protein is critical for SARS-CoV-2 cell entry and infection [2]. Since SARS-CoV-2 was first detected in humans, many mutations have been found in the SARS-CoV-2 genome, and as the virus evolves, many new variants are being found, which affects transmissibility or virulence, disease severity, risk of reinfection, and diagnosis and vaccine performance [3].COVID-19 leads to a range of functional alterations in affected individuals. The respiratory system is primarily affected, with manifestations including acute respiratory distress syndrome (ARDS), pneumonia, and impaired lung function. However, SARS-CoV-2 affects multiple organ systems beyond the respiratory system, such as the cardiovascular, gastrointestinal, renal, and neurological systems. COVID-19-associated functional alterations include myocardial injury, coagulopathy, gastrointestinal symptoms, acute kidney injury, and neurological manifestations. These systemic effects contribute to the complexity and severity of the disease. Understanding the diverse functional alterations caused by COVID-19 is crucial for developing comprehensive management strategies and optimizing patient care [4,5,6].

Nearly 80% of patients affected by COVID-19 have only mild symptoms, recovering with conventional medical treatment or even without any treatment [7,8]. Around 20% of affected patients, however, develop respiratory distress, requiring oxygen therapy or even mechanical ventilation, and nearly 10% of them must be admitted to intensive care units (ICUs) [9]. Moreover, the mortality of late-stage ARDS precipitated by COVID-19 is remarkably high. Around 48–90% of patients intubated and placed on mechanical ventilation do not survive; this percentage is significantly higher than that associated with intubation for other viral pneumonias, which is nearly 22% [10]. In addition, for non-survivors, the median duration from admission to hospital to death is 10 days [10]. Given the severe contagiousness of SARS-CoV-2, the absence of reliable treatments, the elevated mortality rates observed in critical patients, and the short time span between hospital admission and death, it is essential to prioritize the advancement of novel methods for the early identification of cases at risk of becoming critical and the exploration of new targets for therapeutic interventions.

To date, the lack of effective prognostic markers has constituted one of the challenges of monitoring patients who progress to the severe form of COVID-19, especially when therapy is based on clinical manifestations. Thus, it is of vital importance to ascertain which peripheral markers are related to disease severity and to manage treatment at an early stage.

Alterations of plasma proteins are good indicators of pathophysiological changes caused by several diseases, including viral infections. In this sense, plasma proteomics is widely used for biomarker discovery [11]. So far, only a few studies have performed proteomic profiling of plasma/serum of COVID-19 patients [12,13,14,15,16,17]. Among them, some enrolled only a few COVID-19 patients [13,15,17,18,19,20], and one enrolled patients with no need for hospitalization and compared them with hospitalized patients, without distinguishing the severity of the disease among the hospitalized patients [12]. The study conducted by Overmyer, Shishkova, Miller, Balnis, Bernstein, Peters-Clarke, Meyer, Quan, Muehlbauer, Trujillo, He, Chopra, Chieng, Tiwari, Judson, Paulson, Brademan, Zhu, Serrano, Linke, Drake, Adam, Schwartz, Singer, Swanson, Mosher, Stewart, Coon, and Jaitovich [14] evaluated the plasma samples of 102 and 26 patients who tested positive and negative for SARS-CoV-2, respectively. The positive patients were divided into two severity groups based on whether they were admitted to an ICU. The authors identified proteins and metabolites offering pathophysiological insights into the disease and offered therapeutic suggestions. The main limitation, however, was the lack of association of the omics data with survival, which is the most notable outcome measure [14]. In the study by Filbin, Mehta, Schneider, Kays, Guess, Gentili, Fenyves, Charland, Gonye, Gushterova, Khanna, LaSalle, Lavin-Parsons, Lilley, Lodenstein, Manakongtreecheep, Margolin, McKaig, Rojas-Lopez, Russo, Sharma, Tantivit, Thomas, Gerszten, Heimberg, Hoover, Lieb, Lin, Ngo, Pelka, Reyes, Smillie, Waghray, Wood, Zajac, Jennings, Grundberg, Bhattacharyya, Parry, Villani, Sade-Feldman, Hacohen, and Goldberg [16], the authors longitudinally evaluated plasma proteins in 306 COVID-19 patients and 79 symptomatic controls and deconvoluted these data using published scRNA-seq datasets. Comparing the patients who died to severely ill survivors allowed the authors to identify dynamic immune-cell-derived and tissue-associated proteins related to survival, including exocrine pancreatic proteases. The authors proposed a model in which interactions between myeloid, epithelial, and T cells drive tissue damage.

In this study, we profiled host responses to SARS-CoV-2 by performing a shotgun label-free quantitative proteomic analysis of plasma samples from a cohort of 163 COVID-19 patients admitted to Bauru State Hospital (HEB), Brazil, between 4 May 2020 and 4 July 2020. Plasma samples were collected upon admission, and the patients were divided into three groups based on the course of the disease comprising both survivors (mild and severe patients) and non-survivors (critical patients). The proteomic findings were associated with the disease’s severity and the laboratory findings.

## 2. Materials and Methods

### 2.1. Ethical Aspects

This project was approved by the Ethics Committee of the Bauru School of Dentistry, University of São Paulo (CAAE 31019820.8.0000.5417), upon acceptance by the Nucleus of Teaching and Research of the HEB. A waiver of informed consent was approved by the Ethics Committee.

### 2.2. Study Design and Patients

**Figure 1:**
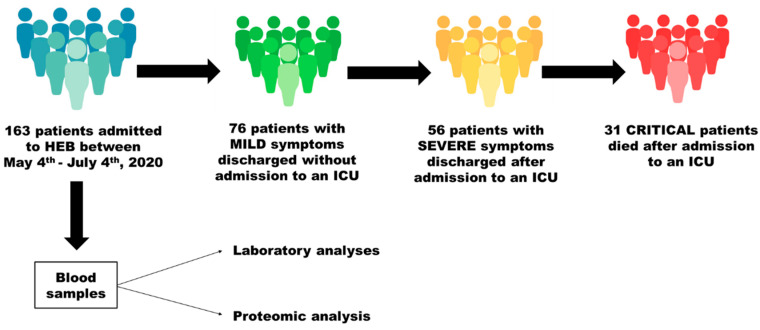
Experimental design of the study.

Figure 1 illustrates the main characteristics of the study along with its cohort design. All patients were admitted to the HEB (Bauru, SP, Brazil) between 4 May and 4 July 2020 and were diagnosed with COVID-19 via RT-PCR of nasopharyngeal swab samples.

All patients received the routine support established by the hospital, which included oxygen support, invasive and non-invasive mechanic ventilation, use of antibiotics, use of vasopressor, use of anticoagulant, renal support therapy, and use of corticoid (if necessary). These patients were all alive upon admission to hospital.

### 2.3. Comparisons and Sampling

The patients were divided into the following 3 groups based on the course of the disease: (a) patients with mild symptoms that were discharged without having been admitted to an ICU; (b) patients with severe symptoms that were discharged after admission to an ICU; (c) critical patients who were admitted to an ICU and died.

Upon admission, blood samples were collected for routine laboratory analyses at the hospital, were analyzed: white blood cell count, neutrophil count, lymphocyte count, eosinophil count, platelet count, hemoglobin, red blood cell count, ferritin, albumin, aspartate aminotransferase (TGO), alanine aminotransferase (TGP), creatinine phosphokinase (CPK), urea, creatinine, C-reactive protein (CRP), lactate dehydrogenase (LDH), and D-dimer. Shortly after collection, an aliquot of blood samples was centrifuged at 2000× *g* for 10 min, and the plasma fraction was stored at −80 °C for proteomic analysis.

### 2.4. Preparation of the Plasma Samples for Proteomic Analysis

Initially, the samples were submitted to depletion, as previously described [21]. For the completion of this process, 60 μL of plasma was diluted in 180 μL of buffer A (Equil/Load/Wash; Agilent, Santa Clara, CA, USA) and vortexed. In sequence, the solution was loaded on Filter Spin (0.22 um; Agilent, Santa Clara, CA, USA) and centrifuged at 16,000× g for 1 min, collected in a tube, and then submitted to Multiple Affinity Removal Column (Agilent Technologies, Santa Clara, CA, USA), according to the manufacturer’s protocol. Buffer A (Agilent, Santa Clara, CA, USA) was used to wash and balance the column, while Buffer B (Agilent, Santa Clara, CA, USA) was employed for the elution of the bound proteins from the column. The low-abundance flow-through fraction was collected and stored at −20 °C for analyses.

In order to facilitate proteomic analyses, it was necessary to exchange the depletome buffer with 50 mM ammonium bicarbonate using Amicon^®^ Ultra 4 mL Centrifugal Filters (Merck, Rahway, NJ, USA). The final volume was 600 µL. The same volume of urea solution (8 mM Urea in 50 mM ammonium bicarbonate buffer) was added. Samples were then quantified [22], and a volume corresponding to 100 μg of proteins was reduced with dithiothreitol (100 mM, 40 °C, and 30 min) and alkylated with iodoacetamide (300 mM, 30 min, room temperature, and in the dark). Then, samples were digested with Pierce™ Trypsin Protease, MS Grade (Thermo Fisher Scientific, Waltham, MA, USA), at a ratio of 2:100 (*w*/*w* trypsin/protein) for 16 h at 37 °C. Digestion was quenched with 5% trifluoroacetic acid (TFA, Sigma-Aldrich Brasil Ltda, Barueri, SP, Brazil) for 15 min at room temperature. The samples were centrifuged at 20,817× *g*, at 6 °C for 30 min. The supernatant was recovered, and the samples were purified and concentrated using Pierce C18 Spin Columns (Thermo Fisher Scientific, Waltham, MA, USA). In sequence, samples were dried and stored for proteomic analyses.

### 2.5. Proteomic Analysis

Shotgun quantitative label-free proteomics was performed in a nanoACQUITY UPLC system (Waters, Milford, MA, USA) coupled with a Xevo Q-TOF G2 mass spectrometer (Waters, Milford, MA, USA), as previously described [23]. Spectra were processed, and proteins were identified and quantified with Progenesis QI for Proteomics^®^ (Nonlinear Dynamics; Waters Corporation; version 4.0) using Apex3D (Waters) for peak detection and searching the Swiss-Prot Human proteomic database, using all the peptides for relative quantification. In order to obtain the preliminary protein dataset, the following parameters were considered: trypsin digestion with a maximum of one missed cleavage; variable modification via oxidation (M) and fixed modification via carbamidomethyl (C); false discovery rate (FDR) less than 4%; and mass error less than 20 ppm. In addition, ion-matching requirements were established to select proteins with at least one ion per peptide, three ions per protein, and one peptide per protein. Then, the final list of proteins was reduced to selected proteins identified by at least two unique peptides and proteins whose presence was detected in at least 60% of the samples.

The software CYTOSCAPE version 3.9.0 was used to build networks of molecular interactions between the identified proteins with the aid of the ClueGo and String applications.

### 2.6. Statistical Analysis

The software GraphPad InStat (version 3.0 for Windows; GraphPad Software Inc., La Jolla, CA, USA) was used. Data were checked for normal distribution using Kolmogorov–Smirnov test and for homogeneity using Bartlett’s test for the selection of the appropriate statistical test. When data passed normality and homogeneity thresholds, they were analyzed via ANOVA and Tukey’s post hoc test. Otherwise, Kruskal–Wallis and Dunn’s tests were used. The significance level, in all cases, was set at 5%. For proteomic analysis, ANOVA was used (*p* < 0.05).

## 3. Results

### 3.1. Characterization of the Patients Included in the Study

The numbers of patients included in each group were 76, 56, and 31 for the mild, severe, and critical patients, respectively, totaling 163 patients (82 men and 81 women). The median age of the critical patients (73.0 years) was significantly higher than that of the mild (51.0 years; *p* < 0.001) and severe (56.5 years; *p* < 0.01) patients, who did not significantly differ from each other in that respect (*p* > 0.05) (Table 1). The percentages of females and males were 53.9/46.1, 53.6/46.4, and 35.5/64.5 for the mild, severe, and critical patients, respectively. The characteristics of the patients in each of the groups regarding comorbidities are summarized in Table 1. 

### 3.2. Laboratory Findings

Regarding the full blood counts, no significant differences were found between the groups in terms of red cells, hemoglobin, and eosinophils. The levels of white cells and neutrophils were significantly higher in the severe and critical patients compared to the mild ones. On the other hand, the levels of lymphocytes were significantly lower in the critical patients compared to mild ones, and platelet levels were significantly lower in the critical patients compared to the mild and severe ones. The other differences were not significant (*p* > 0.05).

As for the biochemical tests, no significant differences between the groups were found regarding TGP levels. Ferritin, TGO, urea, and creatinine levels were significantly higher in critical patients compared to mild and severe ones. Albumin, CPK, LDH, and D-dimer levels were significantly higher in severe and critical patients compared to mild ones. CPR levels were significantly higher in severe patients compared to mild ones. The other differences were not significant (*p* > 0.05).

### 3.3. Proteomic Analysis

**Figure 2:**
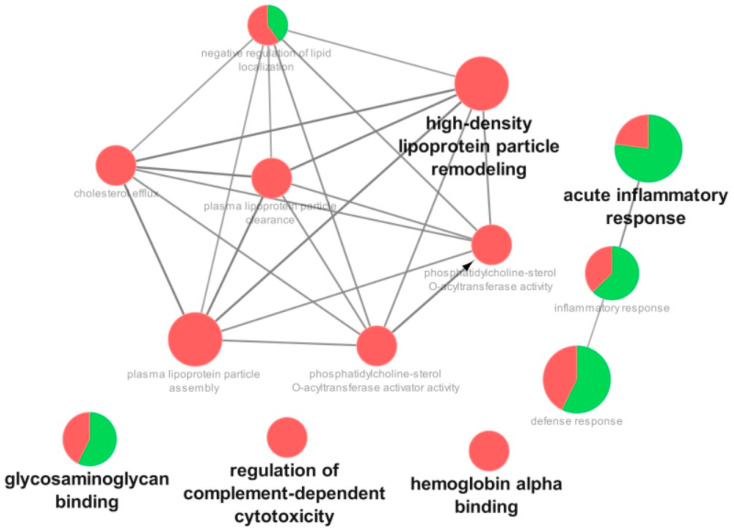
Functional distribution of proteins identified with differential expression in the plasma of patients admitted to Bauru State Hospital, Brazil, between 4 May and 4 July 2020, who were diagnosed with severe or mild COVID-19. Categories of proteins are based on the following GO annotation terms: biological process, immune system, and molecular function. Terms’ significance (Kappa = 0.4) and distribution were determined according to percentages based on the number of associated genes. The green region of the graph indicates upregulated proteins, and the red region indicates downregulated proteins.

**Figure 3:**
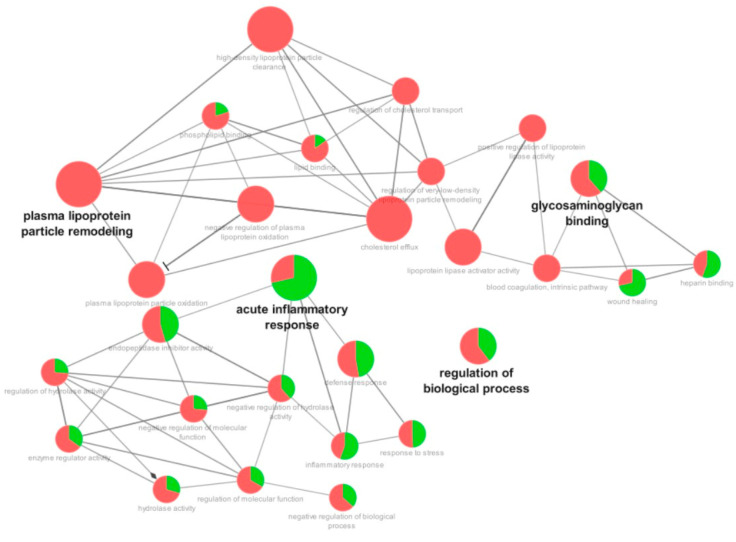
Functional distribution of proteins identified with differential expression in the plasma of patients admitted to Bauru State Hospital, Brazil, between 4 May and 4 July 2020, who were diagnosed with critical or mild COVID-19. Categories of proteins are based on the following GO annotation terms: biological process, immune system, and molecular function. Terms’ significance (Kappa = 0.4) and distribution were determined according to percentages based on the number of associated genes. The green region of the graph indicates upregulated proteins, and the red region indicates downregulated proteins.

**Figure 4:**
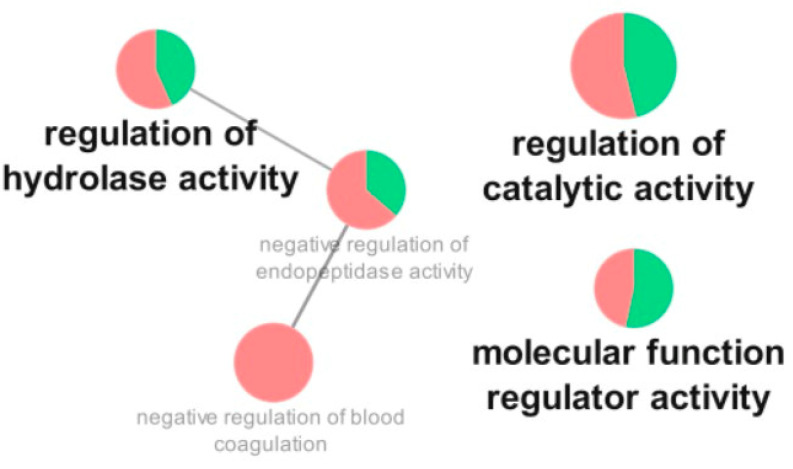
Functional distribution of proteins identified with differential expression in the plasma of patients admitted to Bauru State Hospital, Brazil, between 4 May and 4 July 2020, who were diagnosed with critical or severe COVID-19. Categories of proteins are based on the following GO annotation terms: biological process, immune system, and molecular function. Terms’ significance (Kappa = 0.4) and distribution were determined according to percentages based on the number of associated genes. The green region of the graph indicates upregulated proteins, and the red region indicates downregulated proteins.

Figure 2, Figure 3 and Figure 4 show the functional classifications according to the biological processes, immune system, and molecular function with the most significant terms (bold font) for the following comparisons: severe vs. mild, critical vs. mild, and critical vs. severe, respectively. Regarding the severe vs. mild comparison, the categories with the highest percentages of associated genes were glycosaminoglycan binding (mostly upregulated), regulation of complement-dependent cytotoxicity (downregulated), hemoglobin alpha binding (downregulated), high-density lipoprotein particle remodeling (downregulated), and acute inflammatory response (mostly upregulated) (Figure 2).

Regarding the comparison between the critical vs. mild patients, the most affected categories were plasma lipoprotein particle remodeling (downregulated), acute inflammatory response (mostly upregulated), regulation of biological process (mostly downregulated), and glycosaminoglycan binding (mostly downregulated) (Figure 3). 

As for the comparison of critical vs. severe patients, the most affected categories were regulation of hydrolase activity (mostly downregulated), regulation of catalytic activity (mostly downregulated), and molecular function regulator activity (mostly upregulated) (Figure 4).

**Figure 5:**
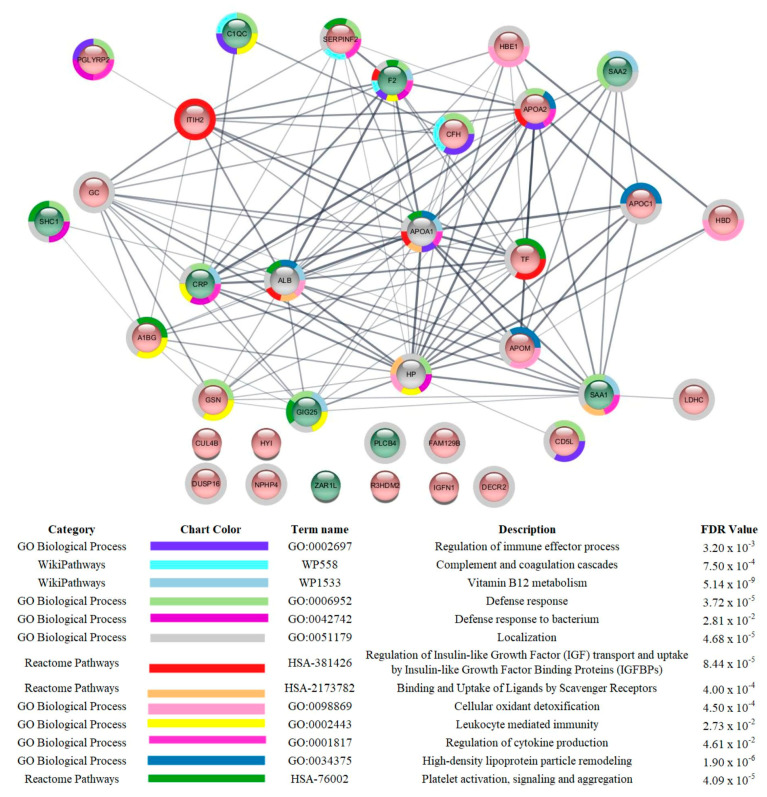
Subnetwork created via String to establish the relationship between proteins identified with differential expression in the plasma of patients admitted to Bauru State Hospital, Brazil, between 4 May and 4 July 2020, who were diagnosed with severe or mild COVID-19. The color of the nodes indicates differences in expression of the respective protein defined by its genes. Light red and light green nodes indicate down- and upregulation in the severe group in comparison to mild. The grey nodes indicate interacting proteins that were offered by CYTOSCAPE but were not identified in the present study.

In the comparison of severe vs. mild patients (Table S1), the levels of 9 proteins were increased and 23 were decreased in severe patients compared to mild ones. The proteins with the highest increases were SHC-transforming protein 1 (SHC1; P29353; 3.47-fold), ZAR1-like protein (ZAR1L; A6NP61; 3.36-fold), Serum amyloid A-1 (P0DJI8; SAA1; 2.41-fold), and Serum amyloid A-2 (P0DJI9; SAA2; 3.32-fold). Other proteins with increased levels were 1-phosphatidylinositol 4_5-bisphosphate phosphodiesterase beta-4 (Q15147; PLCB4), C-reactive protein (P02741; CRP), Alpha-1-antichymotrypsin (P01011; SERPINA3), Complement C1q subcomponent subunit C (P02747; C1QC), and Prothrombin (P00734; F2). The increased proteins in the severe group are mostly related to acute-phase response and immune response. L-lactate dehydrogenase C chain (P07864; LDHC; 2.60-fold) and N-acetylmuramoyl-L-alanine amidase (Q96PD5; PGLYRP2; 2.22-fold) were among the proteins with the highest decreases. Proteins whose levels were decreased but presented lower fold-changes are mainly related to the regulation of complement-dependent cytotoxicity (complement factor H (P08603); CD5 antigen-like (O43866)), the formation of fibrin, coagulation cascades, and plasma lipoprotein particle remodeling (Apolipoprotein C-I (P02654), Apolipoprotein M (O95445), Apolipoprotein A-II (P02652), Alpha-2-antiplasmin (P08697), inter-alpha-trypsin inhibitor heavy chain H2 (P19823), and Alpha-1-B glycoprotein (P04217)). In the interaction subnetwork, proteins with changes in expression interacted mainly with Apolipoprotein A-I (P02647; APOA1), Albumin (P02768; ALB), and Haptoglobin (P00738; HP) (Figure 5).

**Figure 6:**
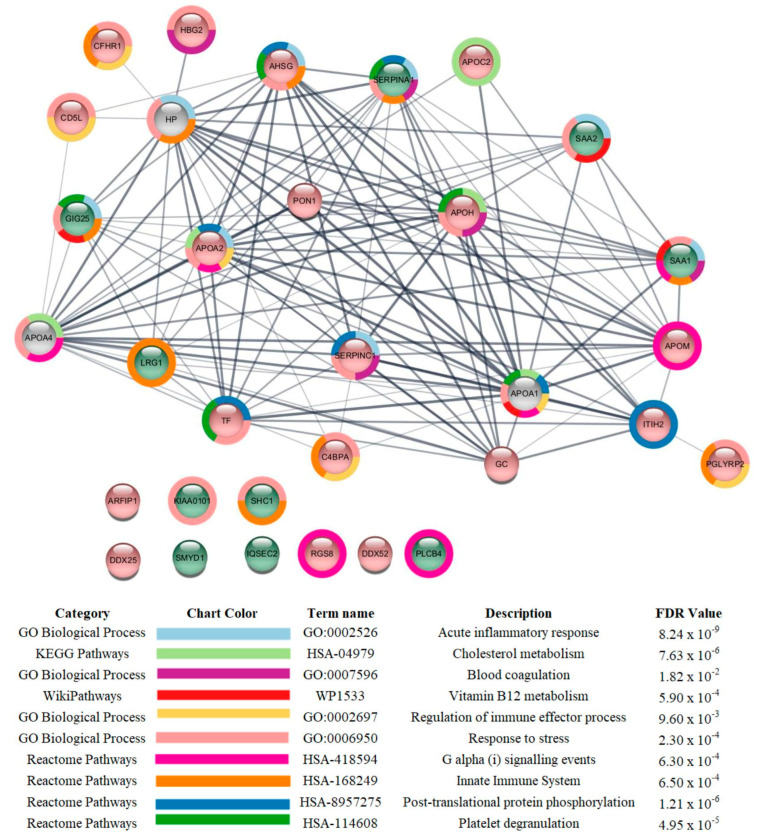
Subnetwork created via String to establish the relationship between proteins identified with differential expression in the plasma of patients admitted to Bauru State Hospital, Brazil, between 4 May and 4 July 2020, who were diagnosed with critical or mild COVID-19. The color of the nodes indicates differences in the expression of the respective protein defined by its genes. Light red and light green nodes indicate down- and upregulation in the critical group with respect to the mild group. The grey nodes indicate interacting proteins that were offered by CYTOSCAPE but were not identified in the present study.

As for the comparison between critical vs. mild patients (Table S2), the levels of 10 proteins were increased and 20 were reduced in the critical patients compared to those with mild symptoms. The proteins with the highest increases were SHC-transforming protein 1 (P29353; SHC1; 4.51-fold), PCNA-associated factor (Q15004; PCLAF; 2.78-fold), Serum amyloid A-2 protein (P0DJI9; SAA2; 2.69-fold), Serum amyloid A-1 protein (P0DJI8; SAA1; 2.51-fold), and 1-phosphatidylinositol 4_5-bisphosphate phosphodiesterase beta-4 (Q151447; PLCB4; 2.19-fold). Other proteins with increased levels were Histone-lysine N-methyltransferase SMYD1 (Q8NB12; SMYD1), Alpha-1-antichymotrypsin (P01011; SERPINA3), Alpha-1-antitrypsin (P01009; SERPINA1), Leucine-rich alpha-2-glycoprotein (P02750; LRG1), and IQ motif and SEC7 domain-containing protein 2 (Q5JU85; IQSEC2). The increased proteins in the critical group are mostly related to acute-phase response, immune response, and the formation of fibrin clots (SAA2 and 1; SERPINA1 and 3; LRG1). Probable ATP-dependent RNA helicase DDX52 (Q9Y2R4; DDX52; 7.26-fold), Arfaptin-1 (P53367; ARFIP1; 2.44-fold) and Apolipoprotein M (O95445;APOM; 2.18-fold) numbered among the proteins with the highest decreases. The proteins whose levels were decreased but that presented lower fold-changes are mainly related to plasma lipoprotein particle remodeling, the formation of fibrin clots, and complement and coagulation cascades, such as Serum paraoxonase/arylesterase 1 (P27169; PON1), Inter-alpha-trypsin inhibitor heavy chain H2 (P19823; ITIH2), Apolipoprotein A-II (P02652;APOA2), Apolipoprotein C-II (P02655;APOC2), Alpha-2-HS-glycoprotein (P02765; AHSG), Antithrombin-III (P01008; SERPINC1), Beta-2-glycoprotein 1 (P02787; APOH); Complement factor H-related protein 1 (Q03591; CFHR1), CD5 antigen-like (O43866), and C4b-binding protein alpha chain (P04003; C4BPA). In the interaction subnetwork, proteins with changes in expression interacted mainly with Apolipoprotein A-I (P02647; APOA1), Apolipoprotein A-IV (P06727; APOA1), and Haptoglobin (P00738; HP) (Figure 6).

**Figure 7:**
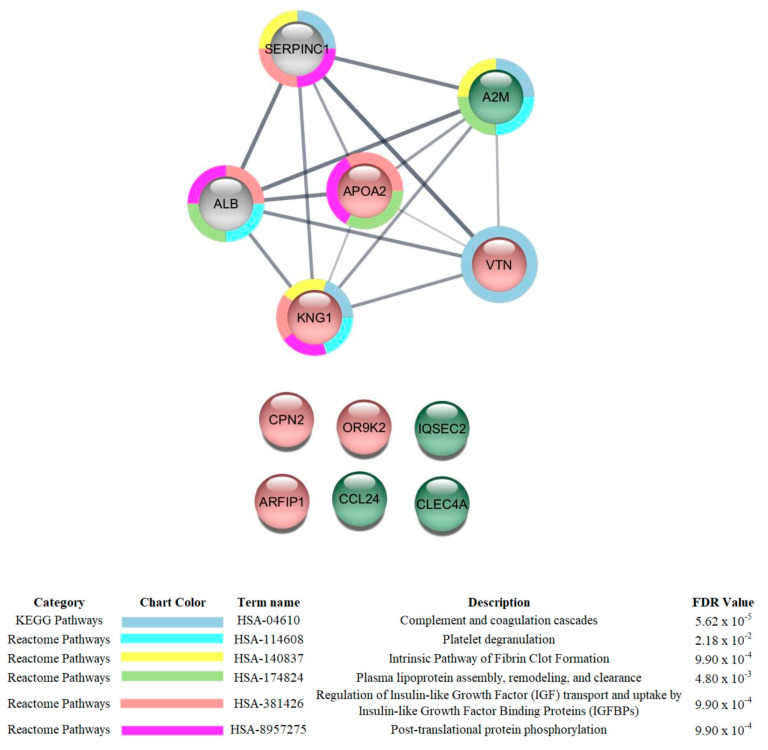
Subnetwork created via String to establish the relationship between proteins identified with differential expression in the plasma of patients admitted to Bauru State Hospital, Brazil, between 4 May and 4 July 2020, who were diagnosed with critical or severe COVID-19. The color of the nodes indicates differences in the expression of the respective protein defined by genes. Light red and light green nodes indicate down- and upregulation in the critical group with respect to the severe group. The grey nodes indicate interacting proteins that were offered by CYTOSCAPE but were not identified in the present study.

When critical patients were compared to severe ones (Table S3), the levels of four proteins were increased and six were decreased in the first group compared to the latter. The protein increases were associated with regulating immune reactivity; cell response and the production of TNF-α (C-type lectin domain family 4 member A; Q9UMR7; CLEC4A and C-C motif chemokine 24; O00175; CCL24); actin cytoskeleton organization and the modulation of chemical synaptic transmission (IQ motif and SEC7 domain-containing protein 2; Q5JU85; IQSEC2); and acute inflammatory response (Alpha-2-macroglobulin; P01023; A2M). Olfactory receptor 9K2 (Q8NGE7; OR9K2; 8.67-fold), which is involved in the activity of the G-protein coupled receptor and in that of the olfactive receptor; Arfaptin-1 (P53367; ARFIP1; 3.78-fold), which regulates protein synthesis; and Carboxypeptidase N subunit 2 (P22792; CPN2; 3.16-fold), which regulates enzymes, numbered among the proteins with the highest decreases. The proteins whose levels were decreased but that presented lower fold-changes are mainly related to acute inflammatory response and cholesterol metabolic processes (Apolipoprotein A-II; P02652; APOA2), blood coagulation and the inflammatory response (Kininogen-1; P01042; KNG), and the immune response (Vitronectin; P04004; VTN). In the interaction subnetwork, proteins with changes in expression interacted mainly with Albumin (P02768; ALB) and Antithrombin-III (P01008; SERPINC1) (Figure 7).

## 4. Discussion

The present study was designed to find out biomarkers of prognosis of COVID-19 patients upon admission to a hospital and to search for possible therapeutical targets. A prospective cohort design was established. All patients admitted to the HEB within a 2-month period were enrolled. Upon admission, we collected blood samples for laboratory and proteomic analyses, and the results were reported according to the course of the disease, for which the patients were classified into three severity categories: mild (survivors who did not need to be admitted to an ICU), severe (survivors who had been admitted to an ICU), or critical (non-survivors who died after admission to an ICU). It is important to mention that some patients seek hospitals at an earlier stage of the disease, while others only do so at an advanced stage of the disease cycle, which makes it more difficult to establish a common analytical framework. So far, only a few studies have performed proteomic profiling of the plasma/serum of COVID-19 patients [12,13,14,15,16,17]. Among these studies, some included a few COVID-19 patients [13,15,17,18,19,20], and one included patients who did not require hospitalization and compared them with hospitalized ones, without distinguishing the severity of the disease among the hospitalized patients [12]. Another study, despite having an adequate sample size and distinguishing the severity of the disease among the hospitalized patients, did not associate omics data with survival [14].

In the present study, the critical patients who died were older than the mild and severe patients, and there was also a predominance of men, thus corroborating previous studies [10,24,25]. The higher susceptibility of males in comparison to females has been attributed to the protective effect of estrogen in women or to stronger immune response with higher levels of cytokines in men [26,27]. The most common comorbidity among the patients was hypertension, followed by diabetes (Table 1), as shown in previous studies [10,24,25]. Alterations in the renin–angiotensin system (RAS), with consequent activation of the NLRP3 (NOD-like receptor 3) inflammasome, is the probable reason why hypertensive patients are more susceptible to severe forms of COVID-19 [28].

In the laboratory exams (Table 2), the plasma hemoglobin levels did not change among the groups, even though the proteomic analysis revealed a reduction in hemoglobin subunit gamma 2 and (critical vs. mild; Table S2) and the hemoglobin subunits epsilon and delta (severe vs. mild; Table S1).

These findings are consistent with those in the literature that report normal, low, or at-the-lower-end-of-the-reference-range hemoglobin levels in COVID-19 patients. Low levels are reported in about 20% of hospitalized patients; they are more common in non-surviving ones and in those presenting hyperinflammation [29]. In the present study, severe and critical patients had increased plasma ferritin levels compared to mild ones, as reported in other studies [30,31,32,33]. Ferritin is a mediator of immune dysregulation. Increased ferritin levels contribute to cytokine storms via direct immune-suppressive and pro-inflammatory effects [34]. Critical and severe patients also presented decreases in serotransferrin (TF; Table S2) levels when compared to mild patients, which is in accordance with findings showing that high ferritin and low transferrin levels are associated with an increased risk for ICU admission and the need for mechanical ventilation [35].

Apolipoprotein A2 (APOA2) levels were reduced in critical and severe patients compared to those presenting mild symptoms (Tables S1 and S2). Moreover, Apolipoprotein C1 (APOC1) levels were reduced in severe compared to mild patients (Table S1), and Apolipoprotein C2 (APOC2) levels were reduced in critical compared to mild (Table S2) patients. These lipoproteins are constituents of HDL and triglyceride-rich lipoproteins such as VLDL. They transport cholesterol from peripheral tissues back to the liver, providing cardioprotective, antiapoptotic, antioxidant, anti-inflammatory, antithrombotic, and anti-infectious functions [36]. Due to these properties, it is not surprising that adequate levels of apolipoproteins are a protective factor for disease severity in COVID-19 infections, as concluded in a recent systematic review [37]. Remarkably, in the present study, APOA2 levels were also reduced in critical patients compared to mild ones (Table S2), which is in agreement with the findings of a systematic review reporting that adequate levels of APOA1 were related to a protection against mortality in patients hospitalized for COVID-19 [37]. These findings indicate that apolipoproteins could be included in the clinical assessment of hospitalized patients with COVID-19, but additional studies are necessary in order to define optimal cut-off points.

Several proteins with expression changes in our study were related to acute inflammatory response and immune response. Our immune system comprises both innate and acquired immune responses. The first causes infected cells to secrete interferons (IFNs) and pro-inflammatory cytokines. IFNs stimulate non-infected cells’ development of an antiviral stage, while proinflammatory cytokines activate macrophages and other phagocytes in order to remove viruses and infected cells. CD4+ Th lymphocytes (adaptive responses) are also activated by cytokines and, in turn, activate B-lymphocytes that will produce neutralizing antibodies to fight the virus and CD8+ Tc cells to initiate the programmed cell death of cells infected by virus. In other words, the innate response exposes the virus and organizes the acquired response to fight the infection. However, the outcome depends on timely coordination between both immune responses. A high production of IFNs 18–24 h post-infection leads to effective innate and acquired immune responses. On the other hand, a delay in the production of IFNs (3–4 days post-infection) results in ineffective innate and acquired immune responses [38], despite persistent IL-6 and TNF-α release by several cells, infiltrating monocytes, inflammatory reactions, and a dysfunctional response amplified by macrophages [39], which may lead to the critical form of COVID-19. In this case, there is a massive increase in cytokine release, which is known as cytokine release syndrome (CRS) or “cytokines storm”. In the present study, the proteins that presented the highest increases in the critical vs. severe patients (Table S2) were CCL24 (C-C motif chemokine 24) and CLEC4 (C-type lectin domain family 4 member A). CLEC4 is a C-type lectin receptor that, once triggered by an antigen, is internalized by clathrin-dependent endocytosis and delivers its antigenic cargo into the antigen presentation pathway, thereby promoting the expansion of CD8+ T cells and high production of IFN-γ and TNFα. CCL24 is related to cellular responses to TNF-α [40]. Functional analyses have revealed the potential role of CLEC4 in viral infection, including COVID-19 [41]. Recently, it was shown that it is possible to predict which COVID-19 patients will clinically deteriorate since they have a blunted IFN and an exaggerated CCL24 airway response [42]. However, the authors mentioned that it is plausible that the identification of inflammatory mediators in the systemic circulation is delayed compared with assessing samples proximal to the site of infection. In our study, blood samples were taken on the day of admission to the hospital; nevertheless, critical patients had increased plasma CCL24 levels compared to severe patients.

In the later stages of inflammation, IL-6 is expressed in the liver, where it elicits the production of acute-phase proteins, such as serum amyloid A1 and A2 (SAA1 and SAA2) [38] and α2-macroglobulin (∝_2_-M) [43,44], whose levels were also increased in critical patients compared to mild ones in the present study (Table S2). SAA levels were also increased in severe patients when compared to mild ones (Table S1). These findings agree with those of previous studies that identified SAA1 and SAA2 as predictors of COVID-19 severity [45,46]. The role of ∝_2_-M in COVID-19 has been proposed based on its versatility; specifically, in its tetrameric form, it is able to inhibit all four classes of proteases, while in its dimeric form, it shows increased interaction with mediators of inflammation, such as TNF-∝, Il-2, and IL-6. Moreover, in children, increased levels of ∝_2_-M are speculated to contribute to the antithrombin activity of plasma and protection against COVID-19, but more studies are necessary to confirm this hypothesis [44]. In the present study, however, ∝_2_-M levels were increased in critical patients compared to severe ones, which suggests its role in inflammation. Another important role of ∝_2_-M—together with ∝_1_-antitrypsin (SERPINA1), whose levels were increased in critical patients compared to mild ones—is the control of neutrophil elastase, which is a key enzyme involved in the formation of NETs (neutrophil extracellular traps) [47]. Extensive formation of NETs, which are web-like protease- and histone-coated DNA structures that constitute an immune mechanism for trapping pathogens, is observed in severe and critical cases of COVID-19 [48] since the alterations can cause platelet activation and thrombosis [49].

The levels of another classical acute-phase protein, C-reactive protein (CRP), were significantly increased in severe patients compared to mild ones in both the proteomic and laboratory analyses (Table S1 and Table 2, respectively). Moreover, CRP levels were increased in critical patients compared to mild ones in both proteomic (Table S2) and laboratory analyses (Table 2). The laboratory analysis validated the results of the proteomic analysis for this protein. Recently, CRP elevation was found to be associated with QTc interval prolongation in patients hospitalized with COVID-19. Prolongation of the QTc interval is associated with an increased risk of ventricular arrhythmias and sudden cardiac death [50]. 

The levels of several proteins associated with immune responses and the complement system were reduced in the severe and critical patients compared to the mild ones (Figure 5 and Figure 6). Among these proteins is CD5 antigen-like (CD5L), which is expressed by macrophages mainly in inflamed tissues and regulates mechanisms in acute or chronic inflammatory responses, as it occurs in infections. It is involved in early responses to microbial infection by acting as a pattern recognition receptor and by promoting autophagy [51]. Vitamin-D-binding protein (VDBP) promotes the enhancement of the chemotactic activity of C5 alpha for neutrophils in inflammation and macrophage activation and for several members of the complement system. α1-B-glycoprotein (A1BG) plays a role in the degranulation of neutrophils and platelets [40]. Furthermore, deceased patients presented decreases in the levels of proteins with important immunomodulatory activities, such as C4b-binding protein alpha chain (C4BPA), complement factor H-related protein 1 (CFHR1), Alpha-2-HS-glycoprotein (AHSG), and N-acetylmuramoyl-L-alanine amidase (PGLYRP2). Reductions in the levels of these proteins might help to explain the altered immune response of these patients that was unable to cope with the viral challenge and its associated consequences.

Hypercoagulability, with a predominance of thrombosis in venous or arterial macro- and microcirculation, worsens prognosis of COVID-19 [49,52,53]. The etiology of COVID-19-associated coagulopathy seems to follow Virchow’s Triad, including abnormalities of blood flow, vascular injury, and abnormalities in the circulating blood [49]. Besides the formation of NETs, other mechanisms are potentially involved in the development of this systemic coagulopathy. Complement-mediated microvascular injury involving lung and skin, with a marked deposition of C5b-9, C4d, and Mannan-binding lectin serine protease (MASP)-2, suggesting the activation of lectin-based and alternative pathways, was reported among autopsy findings from decedents with severe COVID-19 and acute respiratory distress syndrome (ARDS) [54]. In the present study, several proteins related to blood coagulation and platelet degranulation presented changes in expression in critical patients compared to those presenting mild symptoms (Table S2 and Figure 6). Among these proteins were Antithrombin-III (SERPINC1), Beta-2-glycoprotein 1 (APOH), and AHSG, whose levels were decreased in critical patients compared to mild ones. On the other hand, the levels of Alpha-1-antichymotrypsin (SERPINA3) and SAA1 were increased in critical patients in comparison to mild ones. In the present study, D-dimer levels were significantly increased in the plasma of severe and critical patients compared to that of mild ones (Table 2), indicating a state of hypercoagulability in the first two groups. This laboratory result is consistent with the proteomic findings since D-dimer is a fibrin degradation product found in the peripheral blood after fibrin is formed from fibrinogen and degraded by plasminogen activators [49].

Gelsolin (GSN) levels were reduced in severe patients compared to mild ones. This calcium-binding protein scavenges circulating filamentous actin, thus possessing anti-inflammatory properties. For this reason, reduced levels of GSN have been observed in the serum [13,15] and plasma [14] of COVID-19 patients with worse outcomes, constituting a finding that is in agreement with our results. In fact, GSN supplementation has been suggested as a potential therapy for COVID-19 [13], and a clinical trial of recombinant plasma from GSN is currently being conducted (NCT04358406) (Table S1).

The levels of some protective proteins that would be involved in the control of the immune response or blood coagulation were increased in patients with mild symptoms compared to severe (Table S1) and/or critical patients (Table S2). These proteins could potentially help to explain why the disease took a milder course in the former patients. Among the players involved in the immune response is PGLYRP2, which digests biologically active peptidoglycans into biologically inactive fragments [55]. Among the proteins that prevent the formation of clots we found APOH, which prevents the activation of the intrinsic blood coagulation cascade by binding to phospholipids on the surface of damaged cells [40], and Serum paraoxonase/arylesterase 1 (PON1), which possesses aryadialkylphosphatase activity, as it is involved in the protection of low-density lipoproteins against oxidative damage and the subsequent series of events leading to the formation of atheroma [40]. It is also important for the innate immune response, and its levels are reduced during hepatitis virus B infection, correlating with the functional status of the liver [56]. Moreover, in a recent study involving the in silico discovery of candidate drugs against COVID-19, it was reported that genes correlated with ACE2 are enriched in aryadialkylphosphatase activity [57].

Based on our study, potential therapies for COVID-19 should target proteins involved in inflammation, immune responses and the complement system, and blood coagulation, which showed unfavorable alterations in critical and severe patients compared to mild ones. Alterations in some of these proteins have been shown or suggested in cases of COVID-19 in other studies (Table 3).

In addition, other proteins that could be employed in therapies against COVID-19 but that so far have not been associated with the disease are CD5L, VDBP, A1BG, C4BPA, PGLYRP2, SERPINC1, and APOH. The targeting of their pathways may constitute potential new therapies for the disease, which should be evaluated in further studies.

In conclusion, our results have identified several plasma proteins involved in the pathogenesis of COVID-19 that might be useful for predicting the prognosis of the disease when analyzed upon a patient’s admission to a hospital. These proteins are mainly associated with inflammation, immune response/complement system, and blood coagulation. Some of these proteins have already been suggested to be potential biomarkers of prognosis in other studies (Table 4), while others have not. The validation of some of the identified candidates is currently being conducted. Analyses of plasma samples collected from these patients at multiple time points until discharge or death are underway, which will facilitate a more controlled, longitudinal analysis of severity.

## Figures and Tables

**Table 1:** Characteristics * of the patients admitted to Bauru State Hospital, Brazil, between 4 May and 4 July 2020 who were diagnosed with COVID-19 using RT-PCR nasopharyngeal swab samples.

Characteristics	Mild	Severe	Critical
Median age, years (95% CI)	51.0 (48.8–56.7) ^a^	56.5 (51.8–60.4) ^a^	73.0 (63.7–72.7) ^b^
Female (*n*, %)	41, 53.9%	30, 53.6%	11, 35.5%
Male (*n*, %)	35, 46.1%	26, 46.4%	20, 64.5%
Comorbidity			
Hypertension	31.6%	49.1%	64.5%
Diabetes	21.1%	41.8%	32.3%
Cardiovascular disease	10.5%	10.9%	9.7%
Obesity	22.4%	14.5%	3.2%
COPD	5.3%	10.9%	12.9%
Cancer	2.6%	5.5%	3.2%
Nephropathy	3.9%	5.5%	16.1%
Hepatic disease	1.3%	0	0
Stroke	5.3%	1.8%	6.5%
Autoimmune disease	0	0	6.5%

* upon admission. Kruskal–Wallis and Dunn’s test. *n* = 76 (Mild), 56 (Severe), and 31 (Critical). COPD—chronic obstructive pulmonary disease. Patients with mild symptoms were discharged without having been admitted to an intensive care unit (ICU). Patients with severe symptoms were discharged after admission to an ICU. Critical patients died after admission to an ICU. Distinct letters in the same line indicate significant differences among the treatments.

**Table 2:** Laboratory variables ^&^ of patients admitted to Bauru State Hospital, Brazil, between 4 May and 4 July 2020, who were diagnosed with COVID-19 via RT-PCR of nasopharyngeal swab samples.

Characteristics	Mild	Severe	Critical	*p*
*Full blood counts*				
Red blood cells, ×10^6^/mm^3^	4.43 ± 0.58 ^a^	4.24 ± 0.69 ^a^	4.12 ± 0.79 ^a^	0.058 **
White blood cells, /mm^3^	5930 (5565–6949) ^a^	8050 (7090–9090) ^b^	8020 (7349–12024) ^b^	0.001 **
Neutrophil, /mm^3^	4112 (4086–5707) ^a^	6018 (5553–7628) ^b^	5849 (5613 –9889) ^b^	0.005 *
Lymphocyte, /mm^3^	954 (986–1397) ^a^	870 (817–1353) ^a,b^	529 (458–1255) ^b^	0.003 *
Hemoglobin, g/dL	13.0 (12.5–13.8) ^a^	12.8 (12.2–13.2) ^a^	12.5 (11.5–13.2) ^a^	0.217 *
Eosinophil, /mm^3^	0 (15.4–51.8) ^a^	0 (11.7–104.6) ^a^	0 (7.7–61.2) ^a^	0.848 *
Platelets, ×10^3^/mm^3^	220 (210–245) ^a^	220 (207–268) ^a^	176 (154–245) ^b^	0.011 *
*Biochemical tests*				
Ferritin, µg/L	417 (511–796) ^a^	631 (664–1100) ^a^	931 (883–1474) ^b^	0.003 *
Albumin, g/dL	3.60 (3.42–3.82) ^a^	3.20 (3.17–3.41) ^b^	3.1 (2.82–3.23) ^b^	<0.001 *
TGO, U/L	29.5 (31.5–50.0) ^a^	37.0 (36.7–57.1) ^a^	46.0(40.9–81.5) ^b^	0.014 *
TGP, U/L	29.0 (33.5–51.1) ^a^	36.0 (37.6–65.6) ^a^	31.0 (27.8–61.8) ^a^	0.789 *
CPK, U/L	75 (85–138) ^a^	120 (173–344) ^b^	124 (140–865) ^b^	0.001 *
Ure a, mg/dL	32.8 (34.3–50.6) ^a^	33.8 (35.1–54.5) ^a^	44.0 (43.3–60.9) ^b^	0.007 *
Creatinine, mg/dL	0.80 (1.00–1.99) ^a^	0.80 (0.86–1.35) ^a^	1.20 (1.19–4.60) ^b^	0.001 *
PCR, mg/L	46.0 (60.3–93.7) ^a^	120.6 (92.5–140.0) ^b^	102.0 (86.5–151.9) ^a,b^	0.004 *
LDH, U/L	213 (221–263) ^a^	314 (285–363) ^b^	402 (290–531) ^b^	<0.001 *
D–dimer, mg/L	0.73 (0.83–1.32) ^a^	1.11 (1.55–2.62) ^b^	2.14 (1.66–6.43) ^b^	<0.001 *

^&^—at admission. *—Kruskall–Wallis and Dunn´s tests. Data are expressed as median (95% CI). ** ANOVA and Tukey´s tests. Data are expressed as mean ± SD. Patients with mild symptoms were discharged without admission to an intensive care unit (ICU). Patients with severe symptoms were discharged after admission to an ICU. Critical patients died after admission to an ICU. Distinct letters in the same line indicate significant differences among the treatments.

**Table 3:** Potential therapies for COVID-19 based on the main plasma proteomic findings upon admission in the present study and information available in the literature.

Plasma Proteomic Findings	Implications for the Course of COVID-19 According to the Literature	Potential Therapies
GSN levels were reduced in severe patients compared to those with mild symptoms	Calcium-binding protein that scavenges circulating filamentous actin, thus possessing anti-inflammatory properties. Reduced levels of GELS have been shown in serum [13,15] and plasma [14] of COVID-19 patients with worse outcomes.	GSN supplementation has been suggested as a potential therapy for COVID-19 [13], and a clinical trial of recombinant plasma from GSN is currently being conducted (NCT04358406).
PON1 levels were reduced in critical patients compared to those with mild symptoms	This enzyme possesses aryadialkylphosphatase activity, as it is involved in the protection of low-density lipoproteins against oxidative damage and the formation of atheroma [40]. It is also important for the innate immune response [56]. In a recent study involving in silico discovery of candidate drugs against COVID-19, it was reported that genes correlated with ACE2 are enriched in aryadialkylphosphatase activity [57].	Increasing the activity of PON1.
CFHR1 levels were reduced in critical patients compared to those with mild symptoms	Involved in complement regulation. The variant rs414628 found in CFHR1 was associated with severe COVID-19 in adult Caucasian patients [58]	Regulation of CFHR1.
AHSG levels were reduced in critical patients compared to those with mild symptoms	Promotes endocytosis and possesses opsonic properties. This protein was reported to be increased in the serum of survivor COVID-19 patients admitted to the respiratory and ICU because of respiratory failure [59].	Increasing AHSG levels.
SERPINA3 levels were increased in critical and severe patients compared to those with mild symptoms	Inhibits neutrophil cathepsin G and mast cell chymase, both of which can convert angiotensin-1 to the active angiotensin-2. The levels of this protein were reported to be reduced in the serum of survivor COVID-19 patients admitted to the respiratory ward and ICU because of respiratory failure [59] and in the plasma of non-severe compared to severe patients [60]	Inhibition of SERPINA3

**Table 4:** Potential biomarkers of prognosis for COVID-19 based on the main plasma proteomic findings upon admission in the present study and information available in the literature.

Plasma Proteomic Findings	Implications for the Course of COVID-19 According to the Literature
TF levels were reduced in critical and severe patients compared to those with mild symptoms	High ferritin and low transferrin levels are associated with increased risk for ICU admission and the need for mechanical ventilation in COVID-19 patients [35].
APOA1 *, APOA2, APOC1, and APOC2 levels were reduced in critical and/or severe patients compared to those with mild symptoms	Apolipoproteins transport cholesterol from peripheral tissues back to the liver, performing cardioprotective, antiapoptotic, antioxidant, anti-inflammatory, antithrombotic, and anti-infectious functions [36]. Adequate levels of APOA1 were related to protection against mortality in patients hospitalized for COVID-19 [37].
CLEC4 levels were increased in critical patients compared to those with severe symptoms	CLEC4 is a C-type lectin receptor that, once triggered by an antigen, is internalized by clathrin-dependent endocytosis and delivers its antigenic cargo into the antigen presentation pathway, thereby promoting expansion of CD8+ T cells and high production of IFN-γ and TNFα. Functional analysis revealed the potential role of CLEC4A in viral infection, including that of COVID-19 [41].
CCL24 levels were increased in critical patients compared to those with severe symptoms	Chemotactic for resting T-lymphocytes and eosinophils. COVID-19 patients that will clinically deteriorate have a blunted IFN and an exaggerated CCL24 airway response [42].
SAA1 and SAA2 were increased in critical and severe patients compared to those with mild symptoms	SAAs are acute-phase proteins that have been suggested to be predictors of COVID-19 severity [45,46].
CFHR1 levels were reduced in critical patients compared to those with mild symptoms	Involved in complement regulation. The variant rs414628 in CFHR1 was associated with severe COVID-19 in adult Caucasian patients [58]
AHSG levels were reduced in critical patients compared to those with mild symptoms	Promotes endocytosis and possesses opsonic properties. This protein was reported to be increased in the serum of survivor COVID-19 patients admitted to the respiratory and ICU because of respiratory failure [59].
SERPINA3 levels were increased in critical and severe patients compared to those with mild symptoms	Inhibits neutrophil cathepsin G and mast cell chymase, both of which can convert angiotensin-1 to the active angiotensin-2. The levels of this protein were reported to be reduced in the serum of survivor COVID-19 patients admitted to the respiratory and ICU because of respiratory failure [59] and in the plasma of non-severe compared to severe patients [60]

* Interacting protein.

## Data Availability

Not applicable.

## Supplementary Materials

The following supporting information can be downloaded at: https://www.mdpi.com/article/10.3390/cells12121601/s1. Table S1. Proteins with expression significantly altered in the patients with severe symptoms that were discharged after admission to an intensive care unit (ICU) vs. patients with mild symptoms that were discharged without admission to an ICU. Table S2. Proteins with expression significantly altered in the critical patients, who admitted to an intensive care unit (ICU) and died vs patients with mild symptoms that were discharged without admission to an intensive care unit (ICU). Table S3. Proteins with expression significantly altered in the critical patients, who admitted to an intensive care unit (ICU) and died vs patients with severe symptoms that were discharged after admission to an ICU.
