# Supplementary material for: Analysis of Plasma Proteins Involved in Inflammation, Immune Response/Complement System, and Blood Coagulation upon Admission of COVID-19 Patients to Hospital May Help to Predict the Prognosis of the Disease
Source: Cells. 2023 Jun 10;12(12):1601. doi: 10.3390/cells12121601 (PMC10297236; doi:10.3390/cells12121601)
Supplement: Supplementary file 1 [file cells-12-01601-s001.zip › cells-2421590-supplementary.pdf]

Supplementary Tables

Table S1. Proteins with expression significantly altered in the patients with severe symptoms that were discharged after admission to an intensive care unit (ICU) *vs* patients with mild symptoms that were discharged without admission to an ICU.

[a]Acession number	Protein name	Score	[b]*Ratio* 2:1	Anova (p)
P29353	SHC-transforming protein 1	4	3.47	0.03
A6NP61	ZAR1-like protein	9	3.36	0.01
P0DJI8	Serum amyloid A-1 protein	154	2.41	0.00
P0DJI9	Serum amyloid A-2 protein	132	2.32	0.00
Q15147	1-phosphatidylinositol 4_5-bisphosphate phosphodiesterase beta-4	23	1.87	0.00
P02741	C-reactive protein	57	1.79	0.01
P01011	Alpha-1-antichymotrypsin	344	1.39	0.00
P02747	Complement C1q subcomponent subunit C	15	1.33	0.03
P00734	Prothrombin	257	1.24	0.03
P08603	Complement factor H	453	0.88	0.03
P02774	Vitamin D-binding protein	382	0.86	0.04
P19823	Inter-alpha-trypsin inhibitor heavy chain H2	272	0.83	0.00
P02787	Serotransferrin	935	0.83	0.01
P04217	Alpha-1B-glycoprotein	251	0.80	0.03
O43866	CD5 antigen-like	70	0.80	0.01
Q9NUI1	Peroxisomal 2_4-dienoyl-CoA reductase [(3E)-enoyl-CoA-producing]	47	0.79	0.02
Q9Y2K5	R3H domain-containing protein 2	10	0.78	0.04
P02652	Apolipoprotein A-II	137	0.77	0.02
Q86VF2	Immunoglobulin-like and fibronectin type III domain-containing protein 1	32	0.76	0.01
P08697	Alpha-2-antiplasmin	102	0.76	0.03
Q13620	Cullin-4B	6	0.76	0.04
Q5T013	Putative hydroxypyruvate isomerase	15	0.75	0.04
Q9BY84	Dual specificity protein phosphatase 16	14	0.72	0.03
P06396	Gelsolin	60	0.67	0.01
O95445	Apolipoprotein M	11	0.67	0.01
P02100	Hemoglobin subunit epsilon	20	0.63	0.04
O75161	Nephrocystin-4	16	0.60	0.01
P02654	Apolipoprotein C-I	12	0.56	0.01
P02042	Hemoglobin subunit delta	95	0.52	0.04
Q96TA1	Protein Niban 2	5	0.52	0.02
Q96PD5	N-acetylmuramoyl-L-alanine amidase	6	0.45	0.01
P07864	L-lactate dehydrogenase C chain	16	0.38	0.04

[a]Identification is based on proteins ID from UniProt protein database, reviewed only (http://www.uniprot.org/).
[b]Proteins with expression significantly altered are organized according to the ratio.
Group 1: patients with mild symptoms that were discharged without admission to an intensive care unit (ICU)
Group 2: patients with severe symptoms that were discharged after admission to an ICU

Table S2. Proteins with expression significantly altered in the critical patients, who admitted to an intensive care unit (ICU) and died *vs* patients with mild symptoms that were discharged without admission to an intensive care unit (ICU)

[a]Acession number	Protein name	Score	[b]*Ratio* 3:1	Anova (p)
P29353	SHC-transforming protein 1	4	4.51	0.03
Q15004	PCNA-associated factor	5	2.78	0.01
P0DJI9	Serum amyloid A-2 protein	132	2.69	0.00
P0DJI8	Serum amyloid A-1 protein	154	2.51	0.01
Q15147	1-phosphatidylinositol 4_5-bisphosphate phosphodiesterase beta-4	23	2.19	0.03
Q8NB12	Histone-lysine N-methyltransferase SMYD1	10	1.46	0.00
P01011	Alpha-1-antichymotrypsin	344	1.40	0.00
P01009	Alpha-1-antitrypsin	560	1.39	0.00
P02750	Leucine-rich alpha-2-glycoprotein	112	1.36	0.04
Q5JU85	IQ motif and SEC7 domain-containing protein 2	37	1.29	0.03
P04003	C4b-binding protein alpha chain	183	0.86	0.03
O43866	CD5 antigen-like	70	0.84	0.03
Q03591	Complement factor H-related protein 1	28	0.84	0.02
P19823	Inter-alpha-trypsin inhibitor heavy chain H2	272	0.82	0.00
Q9UHL0	ATP-dependent RNA helicase DDX25	11	0.81	0.02
P02787	Serotransferrin	935	0.80	0.01
P57771	Regulator of G-protein signaling 8	8	0.80	0.03
P02749	Beta-2-glycoprotein 1	157	0.78	0.04
P01008	Antithrombin-III	121	0.76	0.04
P02774	Vitamin D-binding protein	382	0.75	0.00
P02765	Alpha-2-HS-glycoprotein	190	0.70	0.01
P69892	Hemoglobin subunit gamma-2	30	0.68	0.03
Q15195	Plasminogen-like protein A	6	0.67	0.01
Q96PD5	N-acetylmuramoyl-L-alanine amidase	6	0.63	0.02
P02655	Apolipoprotein C-II	23	0.60	0.00
P02652	Apolipoprotein A-II	137	0.56	0.00
P27169	Serum paraoxonase/arylesterase 1	22	0.51	0.04
O95445	Apolipoprotein M	11	0.46	0.00
P53367	Arfaptin-1	5	0.41	0.00
Q9Y2R4	Probable ATP-dependent RNA helicase DDX52	4	0.14	0.01

[a]Identification is based on proteins ID from UniProt protein database, reviewed only (http://www.uniprot.org/).
[b]Proteins with expression significantly altered are organized according to the ratio.
Group 1: patients with mild symptoms that were discharged without admission to an intensive care unit (ICU)
Group 3: critical patients, who were admitted to an ICU and died

Table S3. Proteins with expression significantly altered in the critical patients, who admitted to an intensive care unit (ICU) and died *vs* patients with severe symptoms that were discharged after admission to an ICU

[a]Acession number	Protein name	Score	[b]*Ratio* 3:2	Anova (p)
O00175	C-C motif chemokine 24	6	1.58	0.04
Q9UMR7	C-type lectin domain family 4 member A	8	1.49	0.02
Q5JU85	IQ motif and SEC7 domain-containing protein 2	37	1.33	0.04
P01023	Alpha-2-macroglobulin	1155	1.29	0.02
P04004	Vitronectin	152	0.85	0.04
P01042	Kininogen-1	206	0.85	0.04
P02652	Apolipoprotein A-II	137	0.72	0.01
P22792	Carboxypeptidase N subunit 2	5	0.32	0.01
P53367	Arfaptin-1	5	0.26	0.00
Q8NGE7	Olfactory receptor 9K2	5	0.12	0.04

[a]Identification is based on proteins ID from UniProt protein database, reviewed only (http://www.uniprot.org/).
[b]Proteins with expression significantly altered are organized according to the ratio.
Group 2: patients with severe symptoms that were discharged after admission to an ICU
Group 3: critical patients, who were admitted to an ICU and died
